# Supplementary material for: Differences in the predicted nasoseptal flap length among races: A propensity score matching analysis
Source: PLoS One. 2023 Mar 16;18(3):e0283140. doi: 10.1371/journal.pone.0283140 (PMC10019619; doi:10.1371/journal.pone.0283140)
Supplement: Attachment — Submitted filename: Response to reviewers_PONE-D-22-26727.docx [file pone.0283140.s001.docx]

***The pages and lines indicated in the “Response_to_Reviewer” file are displayed based on the revised article with changes highlighted.***

Reviewer #1: This article analyzes the difference of nasoseptal flep length between races and genders which is important during endoscopic sinus surgery. The figures and tables of the article are accurate and the language & article design is understandable.

Reviewer #2: Dear Author,

Thank you for the wonderful work on the NSF. I did enjoy reading it. There are some issues which needs to be addressed, before this article is publication ready. Issues are addressed below:

1. While the authors mention full data availability is provided. No attachment/link is provided as part of the manuscript. Hence it is unknown if full data availability is granted. Please read the terms and conditions regarding PLOS data policy for more information.

**◎ Reply:**

**According to the reviewer’s comment, we added Data Availability Statement. All data in this study are openly available at** [**https://doi.org/10.6084/m9.figshare.21785837.v1**](https://doi.org/10.6084/m9.figshare.21785837.v1)

2. There is systematic error between the word and the reference ([]). There should be a space between the word and the bracket and not immediately bracket. Please fix for all. Please refer to other successful articles published as example of correct citation method.

**◎ Reply:**

**We have corrected the relevant reference.**

3. Need to add comparison between other population data research for same or similar region in the introduction and discussion. The main aim of the paper is to compare between lengths of the of the NSF between race, age and skull. The paper is more related to Forensic Anthropology population data as any measurement involving nasal projection will lead to difference in population. Caucasoid, Mongoloid and Negroid type skull. The reason why you obtain similar values for Caucasians and Middle Eastern is because, they come from the same skull class. Please read and add more to the introduction.

**◎ Reply:**

**The introduction was modified by citing forensic anthropology as an example to assume that there are anatomical differences by race (Page 3, Line 56 to 57).**

4. Make abbreviations available for Table 1 and Figure 2 and 3. For Table 1, PB, SS, SKB abbreviations need to be made available below the text. For Figure 2 and 3, please state all the points used for measurement. Example in Figure 2, the intersection between (A) and (B) is not not mentioned, making the Figure not self-explanatory. Same with Figure 3 at the tip of the nose.
**◎ Reply:**

**Abbreviations are provided below the table. Also, all points used for measurements in Figure 2 and Figure 3 are indicated on the figure.**

5. Methods and materials. While the reason to exclude one data point (East Asian=70) is reasonable when there are limited samples. For this research I would urge maintaining it to 71 because of the 19,650 data of East Asians. There is enough data to choose and run the experiment again. If it were from the Middle Eastern group, then it would be acceptable. I would also recommend deleting the sentence line 74-76 after adding a new data point. Retaining the sentence as it is, does not reflect good diligence.
**◎ Reply:**

**As the reviewer pointed out, East Asian data were sufficient, so the experiment was selected again and corrected to fit 71 people in each group. Page 4, Line 75 to 79 were deleted and Page7, Line 128 to 129 were edited.**

6. Discussion. Similar to point 3, need to add some paragraphs relating to differences in Caucasian, Mongoloid and Negroid-type skull. The reason why there are little references in the discussion is also related to this. The authors need to explore more literature review regarding skull morphology and race. There are some papers which make use of lateral cephalogram articles as well.

**◎ Reply:**

**According to the reviewer's comments, we have added a section on forensic anthropology. Based on forensic anthropological research, we hypothesized that the length of the nasoseptal flap and skull base could differ by race (Page 12 to 13, Line 214 to 227, Line 238 to 239).**
